# Safety and immunogenicity of intranasal parainfluenza virus type 5 (PIV5)–vectored COVID-19 vaccine in adults and teens in an open-label phase 1 trial

**DOI:** 10.1126/sciadv.adw0896

**Published:** 2025-07-04

**Authors:** Paul Spearman, Hong Jin, Peng Xiao, Kristeene Knopp, Henry Radziewicz, Marinka Tellier, Sasha E. Larsen, Bryan J. Berube, Xiao Song, Jamie Kidd, Karnail Singh, Zhuo Li, Maria Cristina Gingerich, Samuel Wu, Susan P. John, Angela Branche, Ann R. Falsey, Rhea Coler, Francois J. Villinger, Biao He

**Affiliations:** ^1^ Infectious Diseases, Cincinnati Children’s Hospital and University of Cincinnati, 3333 Burnet Avenue, Cincinnati, OH 45229, USA.; ^2^ CyanVac LLC, 111 Riverbend Rd, Athens, GA 30602, USA.; ^3^ New Iberia Research Center, University of Louisiana at Lafayette, New Iberia, LA 70560, USA.; ^4^ Center for Global Infectious Disease Research, Seattle Children’s Research Institute, WA 98109, USA.; ^5^ Department of Epidemiology and Biostatistics, College of Public Health, University of Georgia, Athens, GA 30604, USA.; ^6^ Department of Medicine, Infectious Disease Division, University of Rochester, Rochester NY 14642, USA.; ^7^ Department of Global Health and Department of Pediatrics, University of Washington School of Medicine, Seattle, WA 98195, USA.

## Abstract

COVID-19 continues causing substantial mortality despite existing FDA-approved COVID-19 vaccines. An effective COVID-19 vaccine providing durable immunity with minimal reactogenicity is needed. CVXGA1, a PIV5-based intranasal COVID-19 vaccine expressing the Spike (S) protein of SARS-CoV-2, was evaluated in this phase 1 study in adults and teens. CVXGA1 was well tolerated without serious adverse events (AEs) or fever reported. Solicited local and systemic AEs were mostly mild. CVXGA1 elicited S-specific serum and mucosal antibodies and CD8^+^ cytotoxic T lymphocyte responses in all groups. Significantly lower rates of symptomatic COVID-19 infection were reported in groups receiving high-dose CVXGA1 (HD) compared to that in the group receiving low-dose CVXGA1 (LD) during the SARS-CoV-2 delta and omicron waves. The data indicate that CVXGA1 is a potentially effective intranasal COVID-19 vaccine that is immunogenic with minimal reactogenicity.

## Teaser

PIV5-vectored intranasal COVID-19 vaccine is safe and immunogenic in the phase 1 clinical study.

## INTRODUCTION

Severe acute respiratory syndrome coronavirus 2 (SARS-CoV-2) is responsible for COVID-19. During the time of this study from 2021 to 2023, a number of COVID-19 vaccines were available in the US, including mRNA-based vaccines, an adenovirus type 26–vectored vaccine, and an adjuvanted Spike (S) protein–based vaccine. These vaccines were all administered intramuscularly. Waning immunity, emergence of variants evading immunity via antigenic drift, and insufficient mucosal immunity at blocking upper respiratory tract transmission have made these vaccines less effective at reducing symptomatic COVID-19 infection over time (*1*–*4*). In 2024, SARS-CoV-2 still caused significant mortality at a rate that is 1.7% higher than that of influenza virus in the US (*5*). Therefore, there is an urgent need for a next-generation COVID-19 vaccine that is safe and effective with minimal side effects, has long-lasting protection, and is easily administered.

The upper respiratory tract is the initial site of infection by SARS-CoV-2 and the primary site of viral replication. The upper and lower respiratory tracts play a seminal role in the pathogenesis of COVID-19. Blocking viral replication in the upper respiratory tract before progression to the lungs and subsequent pathogenesis is a desirable feature for a prophylactic vaccine that could be achieved with intranasal immunization. The viability of this approach has been previously demonstrated by an intranasal influenza vaccine, the first approved cold-adapted live attenuated influenza vaccine (LAIV; AstraZeneca) approved in the US, which is administered via nasal cavity. Intranasal vaccines can elicit local mucosal immunoglobulin A (IgA) antibodies and stimulate resident immune cells in the respiratory mucosa (*6*). Mucosal immune responses in health care workers against the original SARS-CoV-2 WA.1 strain have been found to correlate with protection against the omicron strain (*7*). Viral-vectored intranasal COVID-19 vaccines based on influenza virus, adenovirus, New Castle disease virus, and respiratory syncytial virus have been evaluated clinically with mixed immunogenicity and efficacy results (*8*), although some have received approval for emergency use authorization (EUA) outside of the US. No intranasal COVID-19 vaccine is now approved in the US.

As an enveloped negative-strand RNA virus in the family Paramyxoviridae, parainfluenza virus type 5 (PIV5) is not known to cause disease in humans (*9*). A PIV5 vector has been extensively studied preclinically for vaccine development against diverse pathogens including Middle East respiratory syndrome coronavirus (*10*) and Rous sarcoma virus (RSV) (*11*). CVXGA1 is a vaccine candidate based on this PIV5 vector that encodes the S protein of SARS-CoV-2 from the ancestral WA.1 strain. Preclinical studies of intranasally administered CVXGA1 have demonstrated its immunogenicity; protective efficacy against viral challenge in mice, hamsters, and ferrets; and ability to block contact transmission in ferrets (*12*, *13*). When used as a booster in hamsters primed with COVID-19 mRNA vaccines, a single intranasal dose of CVXGA1 induced serum cross-neutralizing antibodies against SARS-CoV-2 delta and omicron variants and protected against both homologous and heterologous viral challenge (*12*). In this open-label phase 1 study, we evaluated CVXGA1 for its safety and immunogenicity in healthy people 12 to 53 years of age at two dose levels. Participants’ COVID-19 symptomatic infections were monitored during the study follow-up period.

## RESULTS

### Participants and study conduct

**Fig. 1.**
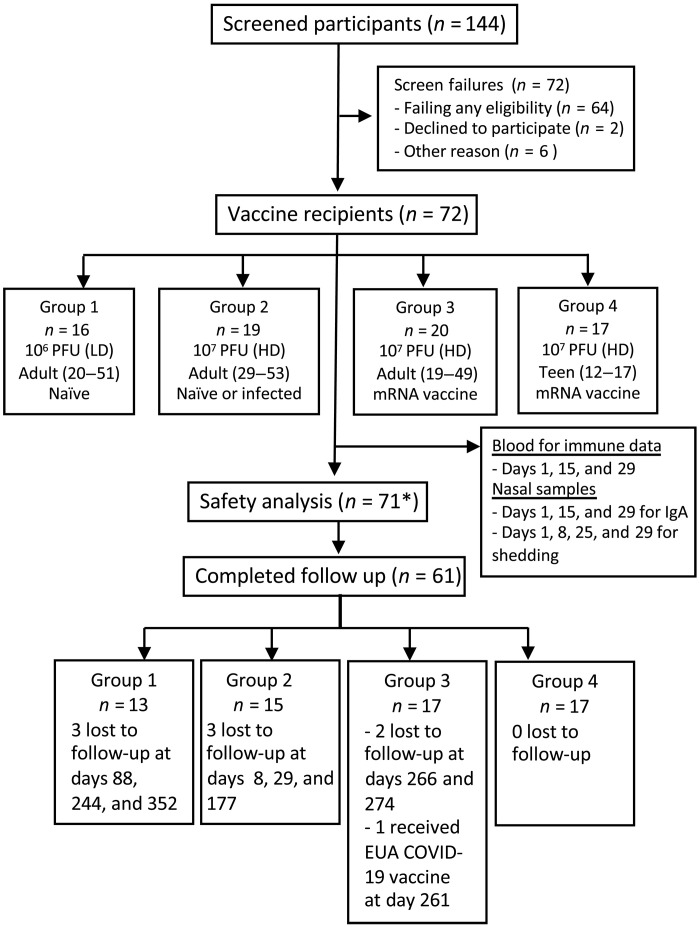
Trial profile. *One participant in group 3 was excluded with COVID diagnosis at day 4.

A total of 72 healthy participants were enrolled in the US over the period from September 2021 to January 2023. Groups 1 to 3 had similar mean ages of 35 to 36 years. The adolescent group 4 had a mean age of 14 years (Table 1). The study originally intended to evaluate low [LD; 10^6^ plaque-forming units (PFU)] and high (HD; 10^7^ PFU) vaccine dose levels in individuals without previous exposure to SARS-CoV-2 or a COVID vaccine (“naïve” participants). During enrollment of group 2 (HD), COVID-19 infection waves in the US made it difficult to enroll naïve individuals, so the protocol was amended to also allow recruitment of participants in group 2 who had exposure to SARS-CoV-2 more than 6 months earlier. Groups 3 and 4 subsequently enrolled adults or adolescents 12 to 17 years of age, respectively, who had prior exposure to SARS-CoV-2 or received an mRNA-based COVID-19 vaccine at least 6 months before intranasal immunization with CVXGA1. Figure 1 outlines the screening and enrollment of participants in this study. There was a balanced distribution between male and female participants, and most participants self-identified as White (Table 1). Ten participants were lost to follow-up during the study with only one occurring before the day 29 visit. The safety analysis consisted of all participants that received one dose of CVXGA1 and completed at least one study follow-up visit (Fig. 1). Immunogenicity analysis excluded seven participants with COVID-19 infection during the first month after receipt of CVXGA1 and those with missing samples.

**Table 1.** Trial cohorts.

Group	1	2	3	4
***N*** ^*^	16	19	20	17
**Dose (PFU)**	10^6^	10^7^	10^7^	10^7^
**Mean age (range years)**	36 (20–51)	36 (25–53)	33 (19–49)	14 (12–17)
**Male/female**	9/7	11/8	10/10	7/10
**Race/ethnicity**				
White	14	11	12	13
Non-white	2	8	8	4
Withdraw number	3	4	3	0
Withdraw window (days)	86–352	8–365	261–274	N/A
Mean days of follow-up†	335	220	261	181

*A total of 72 participants failed screening.

†One participant in group 2 was excluded because of study dropout at day 8, and one participant in group 3 was excluded for COVID-19 positive diagnosis at day 4.

### Safety

No suspected unexpected serious adverse events (SUSARs) related to vaccination were reported. Table 2 summarizes local and systemic reactogenicity and adverse events (AEs) by grade. All solicited local AEs reported were mild (grade 1) or moderate (grade 2) in intensity. Runny nose, reported by 18 participants (25%), was the most solicited local reaction. Sore throat and cough were reported by seven (10%) and nine (13%) participants, respectively. The most common solicited systemic AEs during the first week of vaccination were headache (25%), fatigue (13%), nausea/vomiting (8%), chills (7%), myalgia (6%), and decreased appetite (4%). All were either grade 1 or 2 (Table 2). No fever after vaccination was reported by any participant.

**Table 2.** Solicited local reactogenicity and systemic adverse events. Grading scale: grade 1, mild (awareness of a symptom but the symptom is easily tolerated); grade 2, moderate (discomfort enough to cause interference with usual activity); grade 3, severe (incapacitating; unable to perform usual activities; requires absenteeism from work or bed rest). AE, adverse event.

Solicited local reactogenicity (days 1 to 8): Number (and percentage) of participants reporting grade of event (no grade 3 events reported)^*^			
	Any grade	Grade 1	Grade 2
**Runny nose/nasal congestion**	18 (25%)	18 (25%)	0 (0%)
**Sore throat**	7 (10%)	4 (5%)	3 (3%)
**Cough**	9 (13%)	7 (10%)	2 (3%)
**Solicited systemic AE (days 1 to 8): Number (and percentage) of participants reporting max grade of event (no grade 3 events reported)**			
	**Any grade**		**Grade 2**
**Fever **	0 (0%)	0 (0%)	0 (0%)
**Headache**	18 (25%)	13 (18%)	5 (7%)
**Myalgia**	4 (6%)	4 (6%)	0 (0%)
**Fatigue**	9 (13%)	6 (8%)	3 (4%)
**Chills**	5 (7%)	5 (7%)	0 (0%)
**Nausea/vomiting**	6 (8%)	3 (4%)	3 (4%)
**Decreased appetite**	3 (4%)	2 (3%)	1 (1%)
**Breathing discomfort**	0 (0%)	0 (0%)	0 (0%)

*One participant was diagnosed with COVID-19 on day 4 after vaccination and was excluded from analysis.

### Vaccine virus shedding and genetic stability

On day 2 (1 day postvaccination), vaccine-virus genomic RNA at levels of 4.6 to 5.9 log_10_ copies/ml (estimated viral titer of 2.6 to 3.9 log_10_ PFU/ml) was detected in the nasal swabs of 14 participants who received HD CVXGA1 (groups 2 to 4) but not detected on the next sampling day (day 8). None of the group 1 participants, who received LD CVXGA1, had viral RNA levels above the lower limit of detection (LLOD) of 4.6 log_10_ copies/ml.

Genetic stability of the inserted SARS-CoV-2 S gene and its flanking sequences was evaluated by sequencing reverse transcription polymerase chain reaction cDNAs amplified from nasal swab samples containing viral RNA above the sequencing LLOD of 5.0 log_10_ copies/ml. Only five samples had sufficient RNA levels for sequence information to be obtained, which had the inserted S gene and its flanking gene-start and gene-end sequences identical to the vaccine sequence, demonstrating genetic stability of CVXGA1 in humans.

### Serum antibody responses

**Fig. 2.**
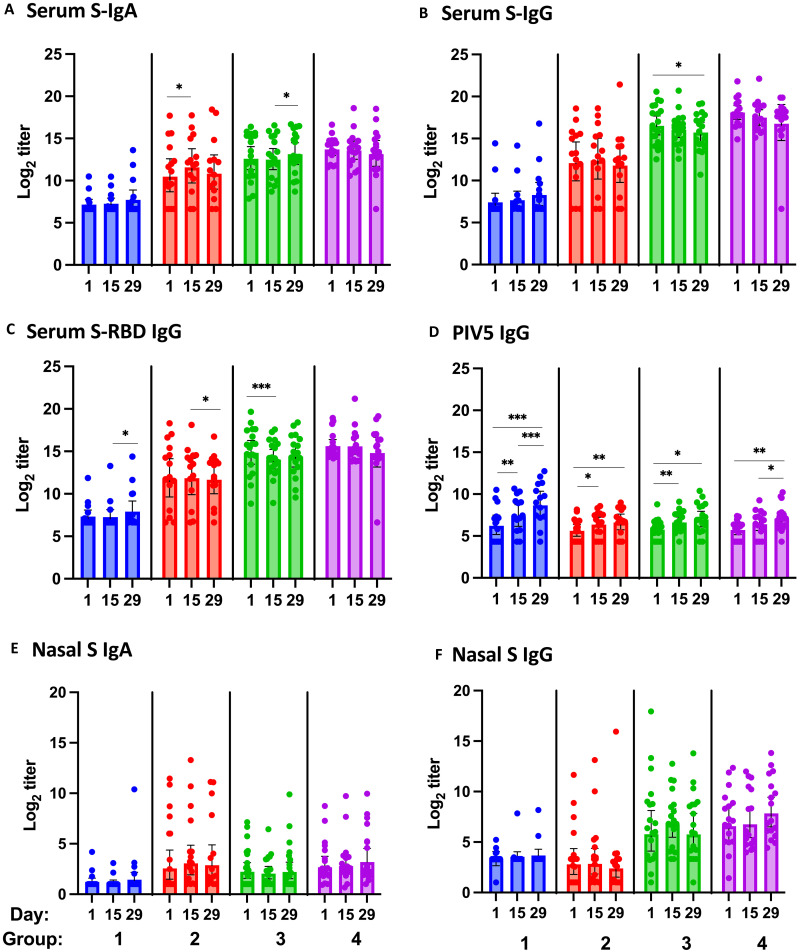
SARS-CoV-2 S-specific antibody and PIV5 vector antibody responses. SARS-CoV-2 S-specific serum IgA (**A**), IgG (**B**), S RBD IgG (**C**), and serum PIV5 antibodies (**D**) and nasal S-specific IgA (**E**) and IgG (**F**) antibodies on days 1 (before vaccination), 15, and 29 for each participant in groups 1 to 4 are presented. GMT with 95% CI indicated. *P < 0.05; **P < 0.01; ***P < 0.005 by Wilcoxon matched-pairs signed-rank test.

Serum S-specific antibody responses measured by S-specific IgA, S-specific IgG, and S-receptor binding domain (RBD)–specific IgG enzyme-linked immunosorbent assays (ELISAs) are shown in Fig. 2. Among all participants in which serum anti-S antibody titers were measured, group 1 (naïve) participants had the lowest baseline antibody titers; LD CVXGA1 increased geometric mean titer (GMT) in this group but not at levels that reached statistical significance. The participants in group 2 who were either naïve (three participants) or previously exposed to SARS-CoV-2 (12 participants) had mean baseline anti-S antibody levels higher than that of group 1 but lower than that of participants who had received COVID-19 mRNA vaccines before CVXGA1 immunization (group 3).

Serum S-specific IgA antibody titer rises postvaccination were detected in all four groups but were statistically significantly higher in group 2 on day 15 versus baseline and group 3 on day 29 versus day 15 (Fig. 2A and fig. S2A). Serum anti-S IgA antibody responses varied among the groups. Serum S-IgG antibody responses were detected in some participants of all groups (Fig. 2B). In group 3, the S-IgG antibody GMT was very high at baseline but significantly declined by day 29. Serum S-RBD–specific IgG antibody responses were statistically higher on day 29 for group 1 (Fig. 2C). Group 3 had higher baseline S-RBD IgG antibody levels than on day 15 postvaccination, and increases in S-RBD–specific IgG antibody responses were not apparent in groups 3 and 4. SARS-CoV-2 neutralizing antibody (nAb) titers against the WA1 strain at baseline had GMTs of 11 (group 1), 66.5 (group 2), 320 (group 3), and 923 (group 4), and nAb titer rises postvaccination were minimal in all groups (Table 3). We noted that one participant in group 4 had their S-specific antibodies dropped to background levels on day 29, which we could not explain. Overall, S-specific serum antibody response rates were similar in the LD group 1 at 35.7% and in the combined HD groups 2, 3, and 4 at 30.8%.

**Table 3.** CVXGA1 elicited antibody and cell-mediated immune (CMI) responses. 5m, 5 months.

Group (participant tested^*^/enrolled)		Group 1	Group 2	Group 3	Group 4
		(*n* = 14 of 16)	(*n* = 16^†^ of 19)	(*n* = 19 of 20)	(*n* = 17 of 17)
Participants	Vaccine dose (PFU)	LD (10^6^)	HD (10^7^ )	HD (10^7^)	HD (10^7^ )
	Mean age, years (range)	36 (20–51)	36 (29–53)	33 (19–49)	14 (12–17)
	Sero-status^‡^	Naïve	Naïve/infected	mRNA vaccine	mRNA vaccine
			5m prior	5m prior	5m prior
Serum antibody responder/total (% responders)	S IgG	5 of 14 (36%)	4 of 15 (27%)	2 of 19 (11%)	3 of 17 (18%)
	S IgA	3 of 14 (21%)	5 of 15 (33%)	4 of 19 (21%)	3 of 17 (18%)
	S RBD IgG	3 of 14 (21%)	2 of 15 (13%)	2 of 19 (11%)	5 of 17 (29%)
	S nAb^‡^	1 of 14 (7%)	2 of 15 (13%)	1 of 19 (5%)	1 of 17 (6%)
	PIV5 IgG	7 of 14 (50%)	6 of 16 (38%)	8 of 18 (44%)	7 of 17 (41%)
**Serum S Ab**	Any S Ab	5 of 14 (35.7%)	5 of 16 (31.3%)	5 of 19 (26.3%)	6 of 17 (35.3%)
Nasal antibody	Nasal S IgG	2 of 14 (14%)	4 of 16 (25%)	9 of 19 (47%)	9 of 17 (53%)
	Nasal S IgA	2 of 14 (14%)	5 of 16 (31%)	6 of 19 (32%)	7 of 17 (41%)
**Nasal S Ab**	IgG and/or IgA	3 of 14 (21.4%)	7 of 16 (43.8%)	10 of 19 (52.6%)	10 of 17 (58.8%)
**Total Ab** ^§^	Any S Ab	6 of 14 (42.9%)	9 of 16 (56.25%)	13 of 19 (68.42%)	11 of 17 (64.7%)
CMI	CD4^+^	7 of 13 (54%)	12 of 12 (100%)	16 of 17 (94%)	15 of 16 (94%)
	CD8^+^CTL	12 of 13 (92%)	12 of 12 (100%)	13 of 17 (76%)	15 of 16 (94%)

*Samples were not tested for participants who became infected with SARS-CoV-2 during the 29 days postvaccination or for which the samples were insufficient. ELISA antibody response rate (serum IgG, IgA, RBD, PIV5, and nasal IgG, IgA, and PIV5 IgG) is defined as >3-fold change on day 15 and/or day 29 over the baseline and nAb against WA.1 as ≥4-fold over baseline (day 1 pre-vaccination) compared to day 15 and/or day 29. CMI response rate is defined as >0.1% rise in at least one cytokine secreting CD4^+^ and CD8^+^ cells each over baseline at day 15 and/or day 29.

†Some were 15 of 19.

‡Seropositive number/total participants at baseline: *n* = 1 of 14 (group 1), 9 of 16 (group 2), 17 of 19 (group 3), and 17 of 17 (group 4).

§Participants with any anti-S antibody-specific response.

### Mucosal antibody responses

Group 1 (naïve) participants had lower baseline nasal IgA antibody levels than participants in the other groups who had prior exposure to SARS-CoV-2 or were immunized with a COVID-19 vaccine (Fig. 2E). Increases in nasal IgA antibody responses were detected in all groups with some participants showing fold rises greater than 10-fold, but GMT change was not statistically significant; individual anti-S nasal IgA titers are shown in fig. S1B. Nasal IgA response rates (greater than threefold increase) were 14% (group 1), 31% (group 2), 32% (group 3), and 41% (group 4) as shown in Table 3. Groups 3 and 4 had higher nasal IgG baselines than groups 1 and 2, presumably reflecting higher IgG antibodies transduced to the mucosa in the recipients previously immunized with mRNA vaccines or by natural infection (Fig. 2F). CVXGA1 intranasal immunization boosted nasal IgG antibody levels in all groups at frequencies between 14 and 53% comparing day 15 and/or day 29 versus baseline depending on cohort composition and dose. Mucosal antibody response rate in the combined HD groups 2, 3, and 4 was 51.9%, which was higher than that of the LD group 1 at 21.4%.

### Overall anti-S antibody response rate

Participants with antibody responses as measured with different assays are summarized in Table 3. For SARS-CoV-2 sero-negative group 1 participants, the highest response rate was seen in serum S-specific IgG antibodies for which 36% of the group demonstrated a response, followed by 21% for serum S-specific IgA antibodies and S-RBD–specific IgG antibodies. Response rates for groups 2 to 4 varied but were mostly in the range of 26 to 35% for any serum S-specific antibody responses. Analysis of the participants with any S-specific antibody responses from serum and/or nasal samples showed that 42.9% (group 1), 56.25% (group 2), 68.42% (group 3), and 64.7% (group 4) responded to CVXGA1 vaccination. Most participants with an anti-S antibody response in more than one assay also had PIV5 antibody responses.

### Preexisting anti-PIV5 immunity and S-specific antibody responses

At baseline, 29% participants had serum anti-PIV5 IgG antibody titers greater than 100. PIV5 vector–specific IgG antibody levels increased postvaccination and were statistically significant on both days 15 and 29 for all groups (Fig. 2D) at rates of 50% (7 of 14), 25% (4 of 16), 22% (4 of 18), and 24% (4 of 17) for groups 1 to 4, respectively. Anti-PIV5 serum antibody levels at baseline versus anti–S-specific antibody responses were evaluated for all groups. Serum anti-S IgA and nasal anti-S IgA response rates based on PIV5 IgG antibody status at baseline are presented in fig. S2 (A and B). The data indicate that preexisting anti-PIV5 antibodies did not negatively affect CVXGA1-induced S-specific antibody responses.

### CMI responses

**Fig. 3.**
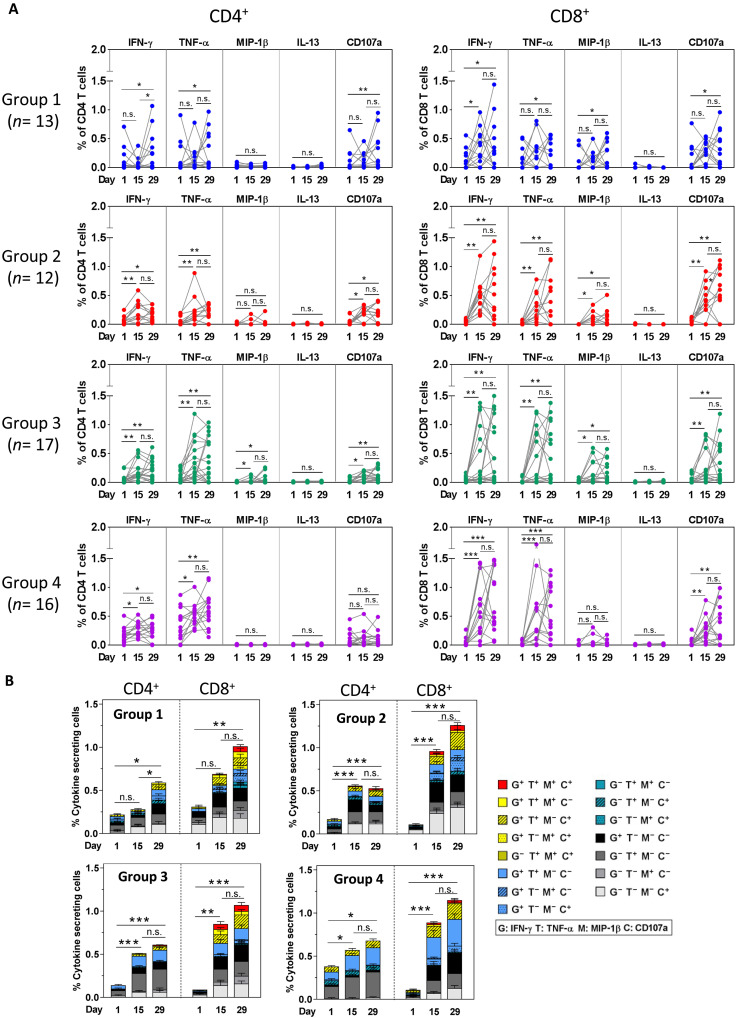
SARS-CoV-2 S-specific CD4^+^ and CD8^+^ T cell frequencies per million PBMCs (by percentage) before and after vaccination. The S peptide–stimulated cells were stained with anti-CD3 Alexa700, anti-CD4 BV605, anti-CD8 BV450, and anti-CD95 PE-Cy5, fixed, and permeabilized. After staining with anti–IFN-γ PE-Cy7, anti–TNF-α APC-Cy7, anti–IL-13 PE, and anti–MIP-1β APC antibodies, the data were acquired on a BD FACSAria Fusion cell sorter. The net percent of CD4^+^ and CD8^+^ cytokine-secreting cells was determined by subtraction of the values obtained with DMSO only stimulated samples (negative control). (**A**) Percentages of CD4^+^ T cells (left) and CD8^+^ T cells (right) of groups 1 to 4 individuals on days 1 (pre-dose), 15, and 29 (represented by symbols and contiguous line) by expression of the indicated immune marker. (**B**) Mean cumulative percentages of CD4^+^ T cells and CD8^+^ T cells of groups 1 to 4 on days 1 (pre-dose), 15, and 29 by expression of the indicated T_H_1/cytotoxic marker combinations (IFN-γ, TNF-α, MIP-1β, and CD107a) by a Boolean combination and SPICE software are represented in stacked histograms. *P < 0.05; **P < 0.01; ***P < 0.005 by Wilcoxon matched-pairs signed-rank test. n.s., not significant.

The S-specific cell-mediated immune (CMI) responses were determined by intracellular cytokine staining (ICS) assay. The participants who had missing peripheral blood mononuclear cell (PBMC) samples or were infected with SARS-CoV-2 during the first 4 weeks after vaccination with CVXGA1 were excluded from sample testing and analysis. Baseline WA.1 S-specific CD4^+^ and CD8^+^ T cells at various levels were detected, with group 1 baselines higher than expected for unknown reasons. Most participants showed increased CD4^+^ or CD8^+^ T helper 1 (T_H_1) or cytotoxic T cell cytokines/markers interferon-γ (IFN-γ), tumor necrosis factor–α (TNF-α), macrophage inflammatory protein–1β (MIP-1β), and CD107a after vaccination. T_H_2 cytokine interleukin-13 (IL-13)–positive T cells were not detected (Fig. 3A). The mean percentages of S-specific CD4^+^ T cells expressing at least one of the T_H_1/cytotoxic markers increased from 0.22, 0.11, 0.14, and 0.38% at baseline to a maximum of 0.59, 0.48, 0.61, and 0.68% 4 weeks after vaccination (*P* < 0.05 or *P* < 0.001; Fig. 3B) for groups 1 to 4, respectively. The mean percentages of the same phenotypes of CD8^+^ T cells increased from 0.31, 0.12, 0.09, and 0.11% at baseline to a maximum of 1.01, 1.26, 1.07, and 1.15% 4 weeks after vaccination (*P* < 0.001) for groups 1 to 4, respectively (Fig. 3B).

S-specific CMI responses to CVXGA1 immunization (defined as an increase from baseline of >0.1% of T cells expressing at least one T_H_1 cytotoxic biomarker for CD4^+^ and CD8^+^ T cells) were calculated (Table 3). CD4^+^ T cell responses were detected in 7 of 13 (54%), 12 of 12 (100%), 16 of 17 (94%), and 15 of 16 (94%) participants for groups 1 to 4, respectively. Mean fold rises of CD4^+^ T cells were 2.68-, 4.36-, 4.35-, and 1.79-fold by day 29 for groups 1 to 4, respectively. S-specific cytotoxic T lymphocyte (CTL) responses (defined as an increase of >0.1% of CD8^+^ T cells expressing at least one cytokine from baseline) were identified in 12 of 13 (92%), 12 of 12 (100%), 13 of 17 (76%), and 15 of 16 (94%) participants for groups 1 to 4, respectively. Mean fold rises of CD8^+^ T cells were 3.26-, 10.50-, 11.89-, and 10.45-fold by day 29 for groups 1 to 4, respectively.

In all groups, the T cells expressing a single T_H_1/cytotoxic biomarker were more frequent than those expressing at least two T_H_1/cytotoxic markers (Fig. 3B). The T cells co-expressing at least two T_H_1/cytotoxic biomarkers (polyfunctional T_H_1/cytotoxic cells) were more frequent in group 4 (teens) versus groups 1 to 3 (adults), reflecting immune system differences in the younger age group. The S-specific CD4^+^ T cells and CD8^+^ T cells expressing type II cytokine IL-13 did not increase after vaccination in any group (Fig. 3A), indicating that there was no T_H_2-biased response to CVXGA1 intranasal immunization.

### Symptomatic SARS-CoV-2 infections during the trial

During the period of the trial from September 2021 to May 2023, various waves of SARS-CoV-2 variant infections emerged in the US. Group 1 (enrolled from September 2021 to February 2022) had the highest overall COVID-19 infection rate: 73.3%, graded as mild or moderate on the AE scale. Groups 2 to 4 had lower infection rates: 11.1% for group 2 (enrolled from February 2022 to November 2022), 22.2% for group 3 (enrolled from April 2022 to September 2022), and 11.8% for group 4 (enrolled from September 2022 to January 2023). All COVID-19 events were graded as mild for groups 2 to 4, except for one episode in a group 4 participant that was graded as moderate on the AE scale. No episodes of hypoxia, lower respiratory tract infection, or hospitalizations related to COVID-19 infection were reported for any participant in the study.

**Fig. 4.**
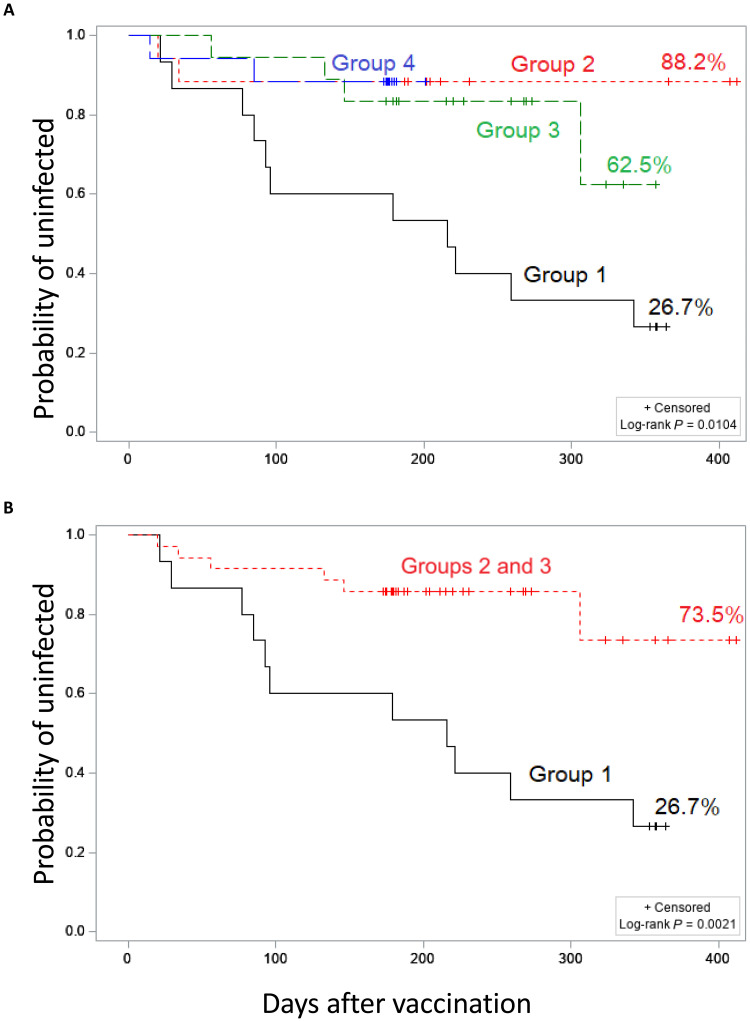
Kaplan-Meier estimates of the probability of remaining uninfected (no symptomatic COVID) over time after receipt of CVXGA1. (**A**) Estimates for groups 1 to 4. (**B**) Estimates for group 1 and combination of groups 2 and 3.

Kaplan-Meier analysis was used to estimate the probability of remaining uninfected (no symptomatic COVID-19 infection) after receipt of CVXGA1 over time for groups 1 to 4 (Fig. 4A). The log-rank test indicated a significant difference in COVID infections among the four groups (*P* = 0.0104). While the age distributions of groups 2 and 3 were similar, group 3 had a lower probability of COVID infection (62.5%) compared to group 2 (88.2%); this may have been due to different exposure to COVID variants or vaccination history as they were enrolled over different time periods. A Kaplan-Meier analysis was also used to examine the probability of remaining uninfected over time for groups 2 and 3 combined, representing the adult target population for COVID booster vaccines (Fig. 4B). Group 4 was excluded from this analysis due to the difference in age of the participants compared with groups 1 to 3. The log-rank test indicated a significant difference between group 1 and groups 2 and 3 combined (*P* = 0.0021). This shows that participants that received HD CVXGA1 (groups 2 and 3) had a significantly lower probability of becoming symptomatically infected with SARS-CoV-2 than participants that received LD CVXGA1 (group 1) with different infection or vaccination history.

Relative vaccine efficacies (rVE) of the HD vaccine (as assessed for groups 2 and 3 combined, group 2 alone, and group 3 alone) versus the LD vaccine (administered to group 1) were also estimated by fitting two separate Cox models adjusted for the time-dependent population infection rate (Table 4). The HD vaccine was estimated to be 67.8% [confidence interval (CI), 1.1 to 89.9%; *P* = 0.048] more effective than the LD vaccine, based on comparison of infection rates of groups 2 and 3 combined versus the infection rate of group 1. Groupwise, the HD vaccine administered to groups 2 and 3 was estimated to be 75.9% (CI, −2.3 to 96.5%; *P* = 0.053) and 61.3% (CI, −30.2 to 90.0%; *P* = 0.126) more effective, respectively, than the LD vaccine. The lack of statistical significance for rVE of the HD vaccine as assessed for group 2 alone and group 3 alone was due to the small sample size.

**Table 4.** Relative vaccine efficacy (rVE) among groups.

Groups	rVE	Confidence interval	*P* value
**Group 2 versus group 1**	75.9%	CI, −2.3 to 96.5%	*P* = 0.053
**Group 3 versus group 1**	61.3%	CI, −30.2 to 90.0%	*P* = 0.126
**Groups 2 and 3 versus group 1**	67.8%	CI, 1.1 to 89.9%	*P* = 0.048

## DISCUSSION

In this first-in-human study, we have demonstrated that the PIV5-vectored COVID-19 intranasal CVXGA1 vaccine is well tolerated in healthy people 12 to 53 years of age and capable of priming and boosting S-specific humoral responses, eliciting robust cell–mediated immune responses as well as local nasal IgA antibody responses. Transient self-limited vaccine shedding was detected in ~25% of participants who received HD CVXGA1 but none in the LD CVXGA1 recipients, indicating that PIV5-vectored vaccine exhibits dose-dependent replication in the human nasal mucosa. Consistent with detection of CVXGA1 replication in HD groups, higher levels of mucosal S-specific antibody response rates were observed in the HD groups (52%) than in the LD group (21%). PIV5-vectored COVID-19 vaccine was genetically stable in humans, like what was reported in a clinical study of a similar PIV5-vectored vaccine targeting RSV (*14*).

Although only a modest serum antibody response was seen for all groups, with overall response rates of 35.7% in the LD (10^6^ PFU) naïve group (group 1) and 26.3 to 35.3% in the HD groups (groups 2 to 4; average 31%), these responses (>3-fold increases in humoral antibody titers) were elicited with a single dose of CVXGA1 despite high levels of anti-S antibodies at baseline in groups 2 to 4 (Fig. 1). The antibody response level detected in CVXGA1-immunized seropositive participants agrees with the serum RSV F IgG antibody response of 21% in RSV sero^+^ adults in the phase 1 study, but the RSV nAb response was ~50% in that study (*14*). Measuring mucosal antibodies can be challenging, and, to measure the absolute amount of S-specific nasal IgA, a qualified assay for S-specific nasal IgA was used. The nasal IgA antibody response was dose dependent, with 14% of participants in group 1 and 31 to 41% in groups 2 to 4, developing nasal IgA responses following vaccination (defined as a >3-fold increase in absolute amount of S-specific IgA over baseline value) regardless of prior vaccination or infection status. Because nasally secreted IgA has been shown to have greater avidity and increased neutralization activity compared with nasal IgG (*15*), this finding is likely relevant for the success of a future intranasal vaccine. CVXGA1 elicited potent S-specific CD8^+^ CTL immune responses in peripheral blood in about 92% (LD group) and 89% (HD groups) of participants, an important immune protection mechanism for vaccines in general.

During this clinical study period, COVID-19 pandemic was causing significant morbidity and mortality, and, because COVID-19 vaccines were available under EUA by the Food and Drug Administration (FDA), it was deemed unacceptable to include a placebo control group. Thus, this study did not directly examine contribution of HD CVXGA1 alone in preventing symptomatic COVID-19 infection in unvaccinated or previously immunized individuals. During the trial period, when different SARS-CoV-2 variants caused waves of infection in the US, Link-Gelles *et al.* (*16*) estimated absolute vaccine efficacy (aVE) of bivalent mRNA vaccines containing ancestral and Omicron S mRNA in those who had received more than two doses of the approved monovalent COVID-19 vaccine, including historically infected individuals. They concluded that bivalent mRNA vaccine had 30% rVE (protection over previously infected and/or vaccinated) and 43% aVE (protection over previously uninfected or vaccinated) as a booster with a median time to infection of 1 month (range, 0 to 2 months) in adults aged 18 to 49 years after immunization during the Omicron BA.4/5 outbreak. Palmer *et al.* (*17*) reported an infection rate of 47% (eight infections with 17 participants > 60 years old) in previously vaccinated adults within 6 months of booster immunization with a self-amplifying RNA-based COVID-19 vaccine. In our study, groups 2 to 4 had very low COVID-19 infection rates after CVXGA1 immunization. We have estimated that the rVE of HD CVXGA1 (as administered to groups 2 and 3 combined) was 67.8% (CI, 1.1 to 89.9%; *P* = 0.048) over LD CVXGA1 (administered to group 1) at 7 months after immunization. If we assume that the group 1 participants derived no protection from LD CVXGA1 and were therefore similar to a placebo group, then the aVE of HD CVXGA1 over placebo control would be 67.8%. To note, the HD groups included previously infected and/or immunized participants, so this estimated aVE would be the result of a combination of prior infection and/or immunization plus HD intranasal CVXGA1, assessed at 7 months after CVXGA1 immunization. Similarly, Link-Gelles *et al.* (*16*) estimated the aVE of mRNA-based bivalent COVID-19 vaccine administered to adults aged 18 to 49 years that had been previously immunized and/or infected, arriving at an aVE of 43% in adults aged 18 to 49 years with a median time to infection of 1 month (range, 0 to 2 months). A key limitation for our study is the small number of participants and lack of placebo control; therefore, a randomized, placebo-controlled clinical study in a larger number of participants, who have had previous COVID infections and/or immunizations (e.g., a combination of groups 2 and 3 in our study), is warranted to confirm the preliminary efficacy results generated in this phase 1 study.

The correlates of protection for a SARS-CoV-2 intranasal vaccine have not been established. It has been shown that nasal IgA and CMI responses correlate with vaccine efficacy for intranasal LAIV (FluMist) (*18*, *19*). While LAIV and trivalent inactivated vaccine (TIV) elicited similar serum antibody responses in children, only LAIV-induced T cell responses were relevant for broadly protective heterosubtypic immunity (*20*). CVXGA1 elicited modest levels of serum antibody responses and dose-dependent mucosal IgA antibody responses, as well as high rates of CD8^+^ cellular immune response. The estimated protection observed in groups 2 to 4 could be a combination of the intranasal vaccine–induced mucosal antibody, serum antibody, CD4^+^ T cells, and CD8^+^ CTL responses. That LD and HD groups had similar CD8^+^ CTL responses, serum antibody response is not considered a determining factor of efficacy for intranasal vaccination, and HD groups (52%) had much higher mucosal antibody responses than LD group (21%) suggests that the 67.8% rVE of HD CVXGA1 over LD CVXGA1 may be attributed to the higher levels of mucosal antibody responses in HD CVXGA1 recipients; i.e., mucosal antibody response may be a correlate of protection for an intranasal COVID-19 vaccine.

To our knowledge, four vectored intranasal COVID-19 vaccines have received authorization outside of the US. An adenovirus type 5 (Ad5)–vectored COVID-19 aerosolized vaccine was approved in China and showed boosting effects in participants previously immunized with inactivated SARS-CoV-2 vaccine (*21*, *22*). Influenza virus–vectored COVID-19 vaccine expressing SARS-CoV-2 S-RBD was approved under EUA in China, although it showed weak immune responses, with 10% serum antibody, 12% nasal IgA antibody, and 46% antigen-specific T cell responses after two doses in phase 2 trials (*23*). An Ad5- and Ad26-vectored intranasal prime/boost vaccine, Sputnik, was approved in Russia. In addition, a chimpanzee adenovirus–vectored COVID-19 vaccine, iNCOVACC, was approved in India and found to stimulate mucosal IgA (12.3%) responses and CMI responses as assessed by S-specific IFN secreting T cells (*24*). A similarly vectored ChAdOX1 nCoV-19 intranasal vaccine was not immunogenic in naïve or vaccinated humans (*25*). Two HDs [10^8^ egg infectious doses (EID)] of NDV-vectored COVID-19 vaccine administered intranasally generated serum anti-S1 antibody responses in 11% (one of nine) and CD8^+^ T cell responses in 44% (four of nine) of naïve participants in a phase 1 study (*26*). Levels of CVXGA1-elicited antibody and cellular immune responses compare favorably with these COVID-19 intranasal vaccines. A single LD (10^6^ PFU) of CVXGA1 generated serum anti-S antibody responses in 36% and CD8^+^ T cell responses in 92% of naïve participants. Considering that CVXGA1 replicates better in human nasal cavities in HD recipients than in LD recipients, we reason that HD CVXGA1 immunization would likely generate more robust and broader immune responses in naïve populations than LD immunization.

In this study, we evaluated PIV5 vector immunity and showed that, overall, 43% of participants had >4-fold increases in PIV5 IgG antibodies regardless of their baseline PIV5 antibody levels. The baseline PIV5 antibody levels had no direct correlation with S-specific antibody responses or anti-PIV5 vector responses. PIV5 has been used as a veterinary vaccine for several decades and a previous study indicated that ~30% of human participants had low levels of nAb to PIV5 (*27*). PIV5 baseline levels and anti-PIV5 antibody response rates in this CVXGA1 study were similar to those observed with our PIV5-vectored RSV vaccine in a phase 1 clinical study that found that preexisting PIV5 antibodies had no impact on RSV and PIV5 antibody seroconversion (*14*).

In conclusion, CVXGA1 administered as a single intranasal dose was well tolerated and immunogenic in humans with or without prior COVID vaccination or infection. CVXGA1 replicates in human nasal cavities in a self-limiting and dose-dependent manner. CVXGA1 generates a wide spectrum of specific immune responses including mucosal and systemic immune responses (serum antibody and CMI). One limitation of this phase 1 study is the lack of the placebo control. Based on the promising data generated from this phase 1 trial, large and controlled studies (NCT05736835 and NCT06742281) with the potential to protect at mucosal sites and prevent virus spread for COVID-19 have been pursued.

## MATERIALS AND METHODS

### Study design and vaccination

This phase 1 open-label, dose-ranging trial was cleared by the FDA under IND 27418 and was conducted at four study sites in the US under registration NCT04954287. Informed consent was obtained from each participant. The first participant was enrolled in group 1 in September 2021, and the final participant was enrolled in January 2023, with follow-up through July 2023. The primary objective was to evaluate the safety and reactogenicity of CVXGA1 up to 28 days after a single intranasal dose of 10^6^ (LD) or 10^7^ PFU (HD, groups 2 to 4). The secondary objectives were to evaluate the immunogenicity of CVXGA1 and to further evaluate safety for up to 1 year. Participants were recruited in four groups (Table 1 and Fig. 1): group 1, SARS-CoV-2 nucleocapsid protein antibody–negative adults; group 2, adults either seronegative or previously infected with SARS-CoV-2 at least 5 months before enrollment; group 3, adults who had received COVID-19 mRNA vaccines >5 months before enrollment (and if they had prior COVID infection, such infections occurred >5 months before enrollment); and group 4: 12- to 17-year-old adolescents who had received COVID-19 mRNA vaccines >5 months before enrollment (and if they had prior COVID infection, such infections occurred >5 months before enrollment). Eligible participants received intranasal CVXGA1 on day 1 with a 0.25-ml spray to each nostril (total volume, 0.5 ml) using a MAD Nasal Intranasal Mucosal Atomization Device (Teleflex MAD300) and observed for 30 min immediately after dosing. Participants were asked to maintain a memory aid for solicited systemic and local reactions during the week after vaccination. Safety oversight of the study was provided by a safety monitory committee. The study was approved by the Advarra Institutional Review Board (IRB) and by IRBs at each of the participating sites.

### Safety assessments

The safety of CVXGA1 intranasal vaccine was assessed over the period of up to 12 months after vaccination, with study follow-up ranging from 181 days for group 4 to 354 days for group 1 (Table 1). Solicited local and systemic AEs including fever were collected for 7 days after vaccination with use of a memory aid. Solicited local AEs included runny nose, sore throat, and cough. Solicited systemic AEs included oral temperature, headache, myalgia, fatigue, decreased appetite, nausea, vomiting, chills, and breathing discomfort. Unsolicited AEs were collected through 28 days after receipt of vaccine, and serious AEs, AEs of special interest, medically attended AEs, and new-onset chronic medical conditions were collected for the study duration (6 to 12 months of follow-up). AEs were graded for severity according to the US FDA “Guidance for industry: Toxicity grading scale for healthy adult and adolescent volunteers enrolled in preventive vaccine clinical trials” and assessed for relationship to vaccination.

### Vaccine shedding and genetic stability

Vaccine virus shedding was analyzed by reverse transcription quantitative polymerase chain reaction in nasal swabs collected on day 1 (pre-vaccination), and days 2, 8, 15, and 29 after vaccination using a validated assay. A regular size flocked nylon swab was used to collect nasal secretions from one nostril, placed in 1.0 ml of universal transport medium (UTM; Copan) and stored at −80°C until sample testing as described previously (*14*). The assay was validated to have a LLOD of 4.6 log_10_ copies/ml, equivalent to ~2.5 log_10_ PFU/ml. Infectivity assays were not conducted. Any nasal swab shedding samples with vaccine virus RNA genome levels greater than 5.0 log_10_ PFU/ml were assessed for vaccine stability by sequencing the SARS-CoV-2 S gene and its flanking regulatory sequences by the Sanger method (Azenta).

### Assessment of serum and mucosal antibody responses against S-protein and PIV5

Blood and nasal samples were collected at baseline (day 1, before vaccine dosing) and at days 15 and 29 after vaccination. SARS-CoV-2 S-specific serum IgG antibodies, S-specific IgA antibodies, and S-RBD–specific IgG antibodies were determined by qualified ELISA assays using trimeric S protein or S-RBD protein (Institute for Protein Design, University of Washington) as previously described (*28*). The data were analyzed with XLfit software (fit model 208), using four-parameter logistic regression analysis of the serially diluted samples and the optical density signal. The endpoint titer was interpolated at the cutoff, determined by the negative control. Responders for humoral endpoints were defined as those with >3-fold rise between postvaccination samples on days 15 and/or day 29 compared to respective baseline titers. This cutoff was based on assay qualification data that showed that a threefold difference was significant, instead of the fourfold difference originally specified in the protocol. A participant was considered sero-positive at baseline for SARS-CoV-2 if serum anti-S IgG antibody titer was above 100 (2.0 log_10_).

SARS-CoV-2 nAb titers were determined by an 80% plaque reduction method (PRNT80) qualified using the SARS-CoV-2 WA.1 strain in a BSL3 laboratory (*28*). Serum nAb titer was determined as the highest serum dilution that has a value below the PRNT80 threshold using GraphPad Prism software to perform the logistic regression analysis. NAb responses were defined by fourfold rise in postvaccination samples (day 15 and/or day 29) compared to respective baseline titers.

S-specific nasal IgA and IgG antibodies from nasal swabs collected directly into 1.0 ml of UTM samples (groups 1 to 3, wet swab) were serially twofold diluted from 1:2 starting dilution and quantified by the ELISA assay using trimeric S protein. The dry swab samples (group 4 only) were eluted in 300 μl of extraction buffer (EB; phosphate-buffered saline containing 0.02% sodium azide and 0.25 M NaCl) for at least 20 min. After removing swabs, the EB-containing secretions were transferred to a Costar spin-X tube (0.22-μm pore size; Sigma-Aldrich, UK) to elute nasal secretions by centrifugation at 4°C for 5 min at 10,000*g*. Repeat centrifugation with a further 300 μl of EB was performed. Samples were stored at −80°C until evaluation. The S-specific nasal IgG or IgA antibody assays were qualified, and antibody responses were defined by >3-fold titer rise in postvaccination samples compared to that in the baseline samples.

PIV5 vector–specific serum IgG titers were determined by a qualified ELISA assay using PIV5 virus–coated plates. The PIV5 IgG endpoint titer was calculated by 4PL curve fitting and reported as reciprocal dilution (*14*). Serum PIV5 antibody titers greater than 100 were considered seropositive.

### Assessment of S-specific CMI responses

SARS-CoV-2 S-specific CMI was examined by ICS assay using cryopreserved PBMCs isolated from whole blood collected on days 1 (before vaccine dosing), day 15, and day 29. PBMCs were stimulated with a SARS-CoV-2 S peptide pool of WA1 strain (GenScript) at 1 μg/ml following the procedure described previously (*14*).

### COVID symptomatic infection rates and comparison of estimated rVE among groups

A Kaplan-Meier estimate for the probability of remaining uninfected (no symptomatic COVID infection) after receipt of CVXGA1 over time was obtained for each group, and a log-rank test was conducted to compare the uninfected probability distributions among the groups. However, this comparison did not adjust for confounding factors. We were mostly interested in comparing among groups 1 to 3 considering group 4’s age difference, but the groups did not start enrollments simultaneously. Group 1 participants enrolled before enrollment of participants in groups 2 and 3, although the means for time to infection or last day of follow-up were similar for groups 1 to 3 at 203, 210, and 228 days, respectively, and the medians were 216, 189, and 224 days, respectively. The general population infection attack rates varied over time, which can affect the infection risk. To address these confounding factors, a Cox model was used to model the hazard of the time-to-infection from vaccination for groups 1 to 3 adjusted for the weekly infection percentage. Specifically, the US weekly COVID-19 cases from the US Centers for Disease Control (https://covid.cdc.gov/covid-data-tracker) and the corresponding US weekly population from US Census Bureau (www.census.gov/popclock/) were used to calculate the weekly infection percentage. The indicators of the two HD groups 2 and 3 were included as covariates, with the LD group 1 as a reference group, and the weekly infection percentage was included as a time-dependent covariate. Specifically for a participant vaccinated at time *t*_0_, the Cox model assumes that the hazard of infection at *u* days from *t*_0_ depends on the most recent weekly infection percentage at time *t*_0_ + *u*. The rVE of the HD group to the LD group was estimated by one minus the estimated hazard ratio adjusting for the weekly infection percentage. Because the number of events was small, the 95% CI was obtained on the basis of the profile likelihood, and the null hypothesis that the vaccine has no efficacy was tested with the (partial) likelihood ratio test. We also compared the HD group (combination of groups 2 and 3) with the LD group (group 1) using the Cox model adjusted for the time-dependent population infection rate. The analysis was conducted using SAS 9.4.

### Statistical analysis of immune data

Statistical analysis was performed using GraphPad Prism software (version 9). Given the small sample size, statistical analysis was mostly descriptive and summative. *P* values were used to show difference of potential significance at a 0.05 significance level. Comparison of SARS-CoV-2 S-specific CMI responses and antibody responses were evaluated by the Wilcoxon matched-pairs signed-rank test (day 15 or day 29 versus day 1, or day 15 versus day 29).

## References

[1] M. Bergwerk, T. Gonen, Y. Lustig, S. Amit, M. Lipsitch, C. Cohen, M. Mandelboim, E. G. Levin, C. Rubin, V. Indenbaum, I. Tal, M. Zavitan, N. Zuckerman, A. Bar-Chaim, Y. Kreiss, G. Regev-Yochay, Covid-19 breakthrough infections in vaccinated health care workers. N. Engl. J. Med. 385, 1474–1484 (2021). 34320281 10.1056/NEJMoa2109072 PMC8362591

[2] R. Link-Gelles, M. E. Levy, K. Natarajan, S. E. Reese, A. L. Naleway, S. J. Grannis, N. P. Klein, M. B. DeSilva, T. C. Ong, M. Gaglani, E. Hartmann, M. Dickerson, E. Stenehjem, A. B. Kharbanda, J. Han, T. L. Spark, S. A. Irving, B. E. Dixon, O. Zerbo, C. E. McEvoy, S. Rao, C. Raiyani, C. Sloan-Aagard, P. Patel, K. Dascomb, A. C. Uhlemann, M. M. Dunne, W. F. Fadel, N. Lewis, M. A. Barron, K. Murthy, J. Nanez, E. P. Griggs, N. Grisel, M. K. Annavajhala, A. Akinseye, N. R. Valvi, K. Goddard, M. Mamawala, J. Arndorfer, D. H. Yang, P. J. Embi, B. Fireman, S. W. Ball, M. W. Tenforde, Estimation of COVID-19 mRNA vaccine effectiveness and COVID-19 illness and severity by vaccination status during omicron BA.4 and BA.5 sublineage periods. JAMA Netw. Open 6, e232598 (2023). 36920396 10.1001/jamanetworkopen.2023.2598 PMC10018321

[3] P. Tang, M. R. Hasan, H. Chemaitelly, H. M. Yassine, F. M. Benslimane, H. A. Al Khatib, S. AlMukdad, P. Coyle, H. H. Ayoub, Z. Al Kanaani, E. Al Kuwari, A. Jeremijenko, A. H. Kaleeckal, A. N. Latif, R. M. Shaik, H. F. Abdul Rahim, G. K. Nasrallah, M. G. Al Kuwari, H. E. Al Romaihi, A. A. Butt, M. H. Al-Thani, A. Al Khal, R. Bertollini, L. J. Abu-Raddad, BNT162b2 and mRNA-1273 COVID-19 vaccine effectiveness against the SARS-CoV-2 Delta variant in Qatar. Nat. Med. 27, 2136–2143 (2021). 34728831 10.1038/s41591-021-01583-4

[4] D. R. Feikin, M. M. Higdon, L. J. Abu-Raddad, N. Andrews, R. Araos, Y. Goldberg, M. J. Groome, A. Huppert, K. L. O'Brien, P. G. Smith, A. Wilder-Smith, S. Zeger, M. Deloria Knoll, M. K. Patel, Duration of effectiveness of vaccines against SARS-CoV-2 infection and COVID-19 disease: Results of a systematic review and meta-regression. Lancet 399, 924–944 (2022). 35202601 10.1016/S0140-6736(22)00152-0 PMC8863502

[5] I. Griffin, J. King, B. C. Lyons, A. L. Singleton, X. Deng, B. B. Bruce, P. M. Griffin, Estimates of SARS-CoV-2 hospitalization and fatality rates in the prevaccination period, United States. Emerg. Infect. Dis. 30, 1144–1153 (2024). 38781926 10.3201/eid3006.231285 PMC11138987

[6] F. E. Lund, T. D. Randall, Scent of a vaccine. Science 373, 397–399 (2021). 34437109 10.1126/science.abg9857

[7] S. Havervall, U. Marking, J. Svensson, N. Greilert-Norin, P. Bacchus, P. Nilsson, S. Hober, M. Gordon, K. Blom, J. Klingstrom, M. Aberg, A. Smed-Sorensen, C. Thalin, Anti-spike mucosal IgA protection against SARS-CoV-2 omicron infection. N. Engl. J. Med. 387, 1333–1336 (2022). 36103621 10.1056/NEJMc2209651 PMC9511632

[8] A. Alu, L. Chen, H. Lei, Y. Wei, X. Tian, X. Wei, Intranasal COVID-19 vaccines: From bench to bed. EBioMedicine 76, 103841 (2022). 35085851 10.1016/j.ebiom.2022.103841 PMC8785603

[9] N. Chatziandreou, N. Stock, D. Young, J. Andrejeva, K. Hagmaier, D. J. McGeoch, R. E. Randall, Relationships and host range of human, canine, simian and porcine isolates of simian virus 5 (parainfluenza virus 5). J. Gen. Virol. 85, 3007–3016 (2004). 15448364 10.1099/vir.0.80200-0

[10] K. Li, Z. Li, C. Wohlford-Lenane, D. K. Meyerholz, R. Channappanavar, D. An, S. Perlman, P. B. McCray Jr., B. He, Single-dose, intranasal immunization with recombinant parainfluenza virus 5 expressing Middle East respiratory syndrome coronavirus (MERS-CoV) spike protein protects mice from fatal MERS-CoV infection. mBio 11, e00554-20 (2020). 32265331 10.1128/mBio.00554-20 PMC7157776

[11] S. I. Phan, Z. Chen, P. Xu, Z. Li, X. Gao, S. L. Foster, M. N. Teng, R. A. Tripp, K. Sakamoto, B. He, A respiratory syncytial virus (RSV) vaccine based on parainfluenza virus 5 (PIV5). Vaccine 32, 3050–3057 (2014). 24717150 10.1016/j.vaccine.2014.03.049 PMC4039636

[12] A. C. Beavis, Z. Li, K. Briggs, M. C. Huertas-Diaz, E. R. Wrobel, M. Najera, D. An, N. Orr-Burks, J. Murray, P. Patil, J. Huang, J. Mousa, L. Hao, T. Y. Hsiang, M. Gale, S. B. Harvey, S. M. Tompkins, R. J. Hogan, E. R. Lafontaine, H. Jin, B. He, Efficacy of parainfluenza virus 5 (PIV5)-vectored intranasal COVID-19 vaccine as a single dose vaccine and as a booster against SARS-CoV-2 variants. bioRxiv 495215 [Preprint] (2022). 10.1101/2022.06.07.495215.

[13] D. An, K. Li, D. K. Rowe, M. C. H. Diaz, E. F. Griffin, A. C. Beavis, S. K. Johnson, I. Padykula, C. A. Jones, K. Briggs, G. Li, Y. Lin, J. Huang, J. Mousa, M. Brindley, K. Sakamoto, D. K. Meyerholz, P. B. McCray Jr., S. M. Tompkins, B. He, Protection of K18-hACE2 mice and ferrets against SARS-CoV-2 challenge by a single-dose mucosal immunization with a parainfluenza virus 5-based COVID-19 vaccine. Sci. Adv. 7, eabi5246 (2021). 34215591 10.1126/sciadv.abi5246 PMC11057785

[14] P. Spearman, H. Jin, K. Knopp, P. Xiao, M. C. Gingerich, J. Kidd, K. Singh, M. Tellier, H. Radziewicz, S. Wu, M. McGregor, B. Freda, Z. Wang, S. P. John, F. J. Villinger, B. He, Intranasal parainfluenza virus type 5 (PIV5)-vectored RSV vaccine is safe and immunogenic in healthy adults in a phase 1 clinical study. Sci. Adv. 9, eadj7611 (2023). 37878713 10.1126/sciadv.adj7611 PMC10599610

[15] T. Suzuki, A. Kawaguchi, A. Ainai, S. Tamura, R. Ito, P. Multihartina, V. Setiawaty, K. N. Pangesti, T. Odagiri, M. Tashiro, H. Hasegawa, Relationship of the quaternary structure of human secretory IgA to neutralization of influenza virus. Proc. Natl. Acad. Sci. U.S.A. 112, 7809–7814 (2015). 26056267 10.1073/pnas.1503885112 PMC4485102

[16] R. Link-Gelles, A. A. Ciesla, K. E. Fleming-Dutra, Z. R. Smith, A. Britton, R. E. Wiegand, J. D. Miller, E. K. Accorsi, S. J. Schrag, J. R. Verani, N. Shang, G. Derado, T. Pilishvili, Effectiveness of bivalent mRNA vaccines in preventing symptomatic SARS-CoV-2 infection—Increasing community access to testing program, United States, September-November 2022. MMWR Morb. Mortal. Wkly Rep. 71, 1526–1530 (2022). 36454688 10.15585/mmwr.mm7148e1 PMC9721148

[17] C. D. Palmer, C. D. Scallan, L. D. Kraemer Tardif, M. A. Kachura, A. R. Rappaport, D. O. Koralek, A. Uriel, L. Gitlin, J. Klein, M. J. Davis, H. Venkatraman, M. G. Hart, J. R. Jaroslavsky, S. Kounlavouth, M. Marrali, C. N. Nganje, K. Bae, T. Yan, K. Leodones, M. Egorova, S. J. Hong, J. Kuan, S. Grappi, P. Garbes, K. Jooss, A. Ustianowski, GRT-R910: A self-amplifying mRNA SARS-CoV-2 vaccine boosts immunity for ≥6 months in previously-vaccinated older adults. Nat. Commun. 14, 3274 (2023). 37280238 10.1038/s41467-023-39053-9 PMC10242235

[18] N. J. Carter, M. P. Curran, Live attenuated influenza vaccine (FluMist(R); Fluenz): A review of its use in the prevention of seasonal influenza in children and adults. Drugs 71, 1591–1622 (2011). 21861544 10.2165/11206860-000000000-00000

[19] D. F. Hoft, K. R. Lottenbach, A. Blazevic, A. Turan, T. P. Blevins, T. P. Pacatte, Y. Yu, M. C. Mitchell, S. G. Hoft, R. B. Belshe, Comparisons of the humoral and cellular immune responses induced by live attenuated influenza vaccine and inactivated influenza vaccine in adults. Clin. Vaccine Immunol. 24, e00414-16 (2017). 27847366 10.1128/CVI.00414-16 PMC5216430

[20] D. F. Hoft, E. Babusis, S. Worku, C. T. Spencer, K. Lottenbach, S. M. Truscott, G. Abate, I. G. Sakala, K. M. Edwards, C. B. Creech, M. A. Gerber, D. I. Bernstein, F. Newman, I. Graham, E. L. Anderson, R. B. Belshe, Live and inactivated influenza vaccines induce similar humoral responses, but only live vaccines induce diverse T-cell responses in young children. J. Infect. Dis. 204, 845–853 (2011). 21846636 10.1093/infdis/jir436 PMC3156924

[21] J. X. Li, S. P. Wu, X. L. Guo, R. Tang, B. Y. Huang, X. Q. Chen, Y. Chen, L. H. Hou, J. X. Liu, J. Zhong, H. X. Pan, F. J. Shi, X. Y. Xu, Z. P. Li, X. Y. Zhang, L. B. Cui, W. J. Tan, W. Chen, F. C. Zhu, C.-S. G. CanSino, Safety and immunogenicity of heterologous boost immunisation with an orally administered aerosolised Ad5-nCoV after two-dose priming with an inactivated SARS-CoV-2 vaccine in Chinese adults: A randomised, open-label, single-centre trial. Lancet Respir. Med. 10, 739–748 (2022). 35605625 10.1016/S2213-2600(22)00087-X PMC9122540

[22] S. Wu, J. Huang, Z. Zhang, J. Wu, J. Zhang, H. Hu, T. Zhu, J. Zhang, L. Luo, P. Fan, B. Wang, C. Chen, Y. Chen, X. Song, Y. Wang, W. Si, T. Sun, X. Wang, L. Hou, W. Chen, Safety, tolerability, and immunogenicity of an aerosolised adenovirus type-5 vector-based COVID-19 vaccine (Ad5-nCoV) in adults: Preliminary report of an open-label and randomised phase 1 clinical trial. Lancet Infect. Dis. 21, 1654–1664 (2021). 34324836 10.1016/S1473-3099(21)00396-0 PMC8313090

[23] F. Zhu, C. Zhuang, K. Chu, L. Zhang, H. Zhao, S. Huang, Y. Su, H. Lin, C. Yang, H. Jiang, X. Zang, D. Liu, H. Pan, Y. Hu, X. Liu, Q. Chen, Q. Song, J. Quan, Z. Huang, G. Zhong, J. Chen, J. Han, H. Sun, L. Cui, J. Li, Y. Chen, T. Zhang, X. Ye, C. Li, T. Wu, J. Zhang, N. S. Xia, Safety and immunogenicity of a live-attenuated influenza virus vector-based intranasal SARS-CoV-2 vaccine in adults: randomised, double-blind, placebo-controlled, phase 1 and 2 trials. Lancet Respir. Med. 10, 749–760 (2022). 35644168 10.1016/S2213-2600(22)00131-X PMC9135375

[24] C. Singh, S. Verma, P. Reddy, M. S. Diamond, D. T. Curiel, C. Patel, M. K. Jain, S. V. Redkar, A. S. Bhate, V. Gundappa, R. Konatham, L. Toppo, A. C. Joshi, J. S. Kushwah, A. P. Singh, S. Gaidhane, K. S. Vadrevu, and BBV154 Study Group, Immunogenicity and tolerability of BBV154 (iNCOVACC), an intranasal SARS-CoV-2 vaccine, compared with intramuscular Covaxin in healthy adults: A randomised, open-label, phase 3 clinical trial (2023); 10.2139/ssrn.4342771.

[25] M. Madhavan, A. J. Ritchie, J. Aboagye, D. Jenkin, S. Provstgaad-Morys, I. Tarbet, D. Woods, S. Davies, M. Baker, A. Platt, A. Flaxman, H. Smith, S. Belij-Rammerstorfer, D. Wilkins, E. J. Kelly, T. Villafana, J. A. Green, I. Poulton, T. Lambe, A. V. S. Hill, K. J. Ewer, A. D. Douglas, Tolerability and immunogenicity of an intranasally-administered adenovirus-vectored COVID-19 vaccine: An open-label partially-randomised ascending dose phase I trial. EBioMedicine 85, 104298 (2022). 36229342 10.1016/j.ebiom.2022.104298 PMC9550199

[26] S. Ponce-de-Leon, M. Torres, L. E. Soto-Ramirez, J. J. Calva, P. Santillan-Doherty, D. E. Carranza-Salazar, J. M. Carreno, C. Carranza, E. Juarez, L. E. Carreto-Binaghi, L. Ramirez-Martinez, G. Paz De la Rosa, R. Vigueras-Moreno, A. Ortiz-Stern, Y. Lopez-Vidal, A. E. Macias, J. Torres-Flores, O. Rojas-Martinez, A. Suarez-Martinez, G. Peralta-Sanchez, H. Kawabata, I. Gonzalez-Dominguez, J. L. Martinez-Guevara, W. Sun, D. Sarfati-Mizrahi, E. Soto-Priante, H. E. Chagoya-Cortes, C. Lopez-Macias, F. Castro-Peralta, P. Palese, A. Garcia-Sastre, F. Krammer, B. Lozano-Dubernard, Interim safety and immunogenicity results from an NDV-based COVID-19 vaccine phase I trial in Mexico. NPJ Vaccines 8, 67 (2023). 37164959 10.1038/s41541-023-00662-6 PMC10170424

[27] Z. Chen, P. Xu, G. W. Salyards, S. B. Harvey, B. Rada, Z. F. Fu, B. He, Evaluating a parainfluenza virus 5-based vaccine in a host with pre-existing immunity against parainfluenza virus 5. PLOS ONE 7, e50144 (2012). 23185558 10.1371/journal.pone.0050144 PMC3502407

[28] S. E. Larsen, B. J. Berube, T. Pecor, E. Cross, B. P. Brown, B. D. Williams, E. Johnson, P. Qu, L. Carter, S. Wrenn, E. Kepl, C. Sydeman, N. P. King, S. L. Baldwin, R. N. Coler, Qualification of ELISA and neutralization methodologies to measure SARS-CoV-2 humoral immunity using human clinical samples. J. Immunol. Methods 499, 113160 (2021). 34599915 10.1016/j.jim.2021.113160 PMC8481082

