# Supplementary material for: Safety and immunogenicity of intranasal parainfluenza virus type 5 (PIV5)–vectored COVID-19 vaccine in adults and teens in an open-label phase 1 trial
Source: Sci Adv. 2025 Jul 4;11(27):eadw0896. doi: 10.1126/sciadv.adw0896 (PMC12227042; doi:10.1126/sciadv.adw0896)
Supplement: Supplementary file 1 — Figs. S1 and S2 [file sciadv.adw0896_sm.pdf]

Science Advances
AAAS

Supplementary Materials for

Safety and immunogenicity of intranasal parainfluenza virus type 5 (PIV5)–vectored COVID-19 vaccine in adults and teens in an open-label phase 1 trial

Paul Spearman *et al.*

Corresponding author: Paul Spearman, paul.spearman@cchmc.org; Biao He, bh1@cyanvacllc.com

Sci. Adv. **11**, eadw0896 (2025)
DOI: 10.1126/sciadv.adw0896

This PDF file includes:

Figs. S1 and S2

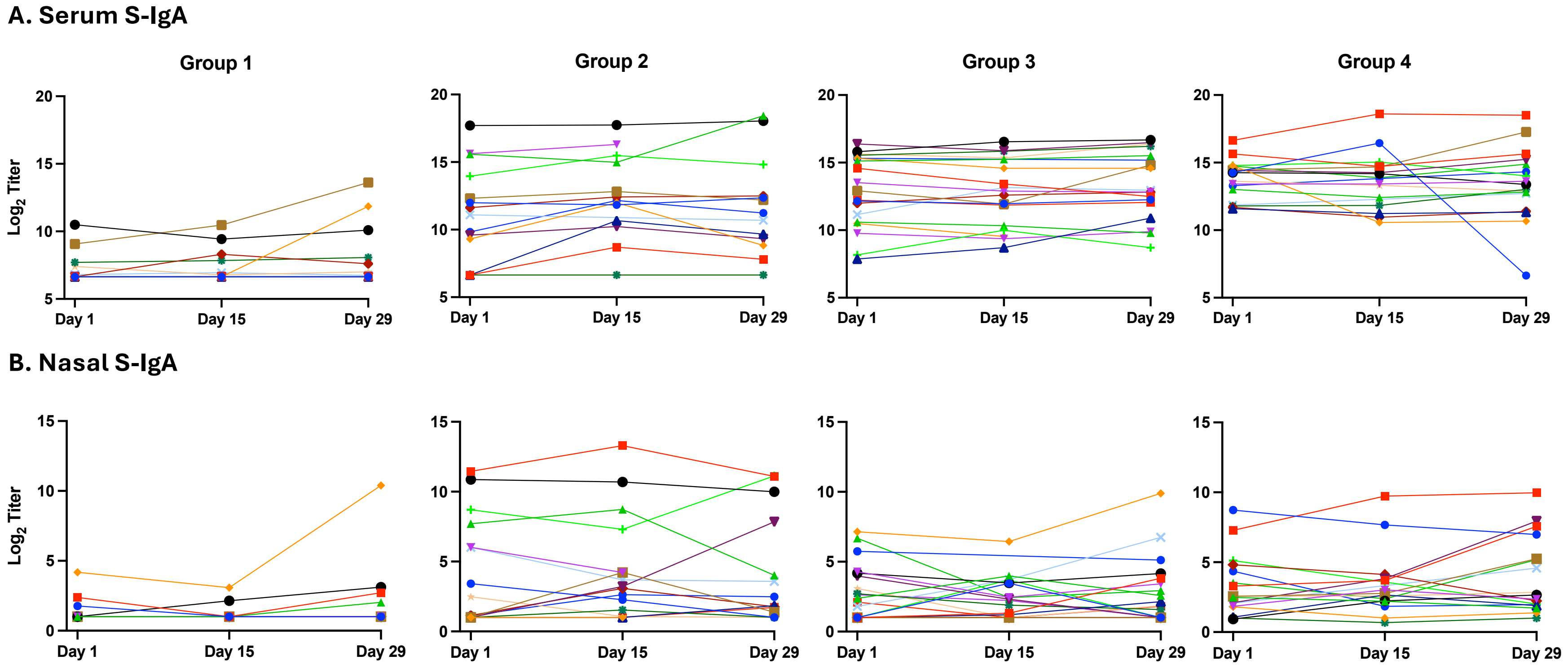

Supplemental Figure 1. Individual serum IgA (**A**) and nasal IgA (**B**) antibody responses on Day 1 (pre-dose) and Days 15 and 29 postvaccination. Symbol colors represent the same subject within a group.

A.

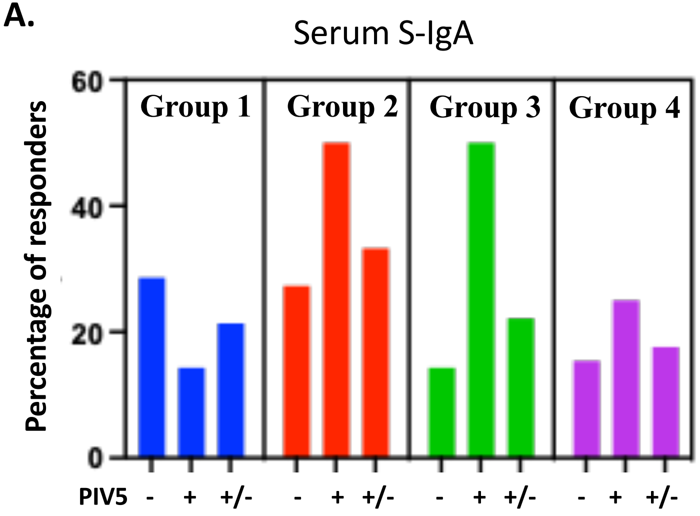

B.

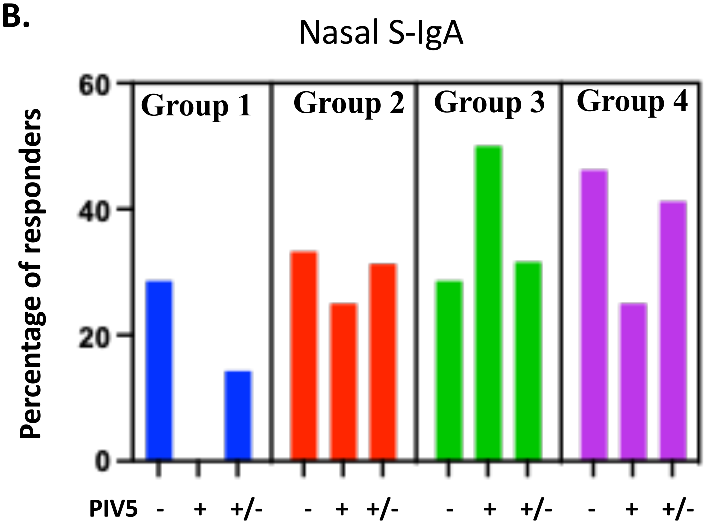

Supplemental Figure 2. Serum IgA (**A**) and nasal IgA (**B**) antibody response rate based on PIV5 seronegative (-) or PIV5 seropositive (+) or total including seronegative and seronegative (+/-) with antibody titer rise of >3-fold on Days 15 and/or Day 29 versus Day 1 (pre-dose).
